# Feasibility and safety study of wearable cyborg Hybrid Assistive Limb for pediatric patients with cerebral palsy and spinal cord disorders

**DOI:** 10.3389/fneur.2023.1255620

**Published:** 2023-11-03

**Authors:** Kazushi Takahashi, Masafumi Mizukami, Hiroki Watanabe, Mayumi Matsuda Kuroda, Yukiyo Shimizu, Takashi Nakajima, Hirotaka Mutsuzaki, Hiroshi Kamada, Kayo Tokeji, Yasushi Hada, Kazunori Koseki, Kenichi Yoshikawa, Tomohiro Nakayama, Nobuaki Iwasaki, Hiroaki Kawamoto, Yoshiyuki Sankai, Masashi Yamazaki, Akira Matsumura, Aiki Marushima

**Affiliations:** ^1^Department of Physical Therapy, Ibaraki Prefectural University of Health Sciences Hospital, Ami, Japan; ^2^Graduate School of Health Science, Ibaraki Prefectural University of Health Sciences, Ami, Japan; ^3^Department of Physical Therapy, Ibaraki Prefectural University of Health Sciences, Ami, Japan; ^4^Department of Neurosurgery, Institute of Medicine, University of Tsukuba, Tsukuba, Japan; ^5^Department of Rehabilitation Medicine, Institute of Medicine, University of Tsukuba, Tsukuba, Japan; ^6^Center for Cybernics Research, University of Tsukuba, Tsukuba, Japan; ^7^Department of Neurology, National Hospital Organization Niigata National Hospital, Kashiwazaki, Japan; ^8^Center for Medical Science, Ibaraki Prefectural University of Health Sciences, Ami, Japan; ^9^Department of Orthopaedic Surgery, Institute of Medicine, University of Tsukuba, Tsukuba, Japan; ^10^Department of Pediatric, Ibaraki Prefectural University of Health Sciences Hospital, Ami, Japan; ^11^Department of Pediatric Neurology, Tsuchiura Rehabilitation Hospital, Tsuchiura, Japan

**Keywords:** Hybrid Assistive Limb, wearable cyborg, gait training, cerebral palsy, spinal cord disorder

## Abstract

**Introduction:**

The wearable cyborg Hybrid Assistive Limb (HAL) is the world’s first cyborg-type wearable robotic device, and it assists the user’s voluntary movements and facilitates muscle activities. However, since the minimum height required for using the HAL is 150 cm, a smaller HAL (2S size) has been newly developed for pediatric use. This study aimed to (1) examine the feasibility and safety of a protocol for treatments with HAL (2S size) in pediatric patients and (2) explore the optimal method for assessing the efficacy of HAL.

**Methods:**

This clinical study included seven pediatric patients with postural and motor function disorders, who received 8–12 sessions of smaller HAL (2S size) treatment. The primary outcome was the Gross Motor Function Measure-88 (GMFM-88). The secondary outcomes were GMFM-66, 10-m walk test, 2- and 6-min walking distances, Canadian Occupational Performance Measure (COPM), a post-treatment questionnaire, adverse events, and device failures. Statistical analyses were performed using the paired samples *t*-test or Wilcoxon signed-rank test.

**Results:**

All participants completed the study protocol with no serious adverse events. GMFM-88 improved from 65.51 ± 21.97 to 66.72 ± 22.28 (*p* = 0.07). The improvements in the secondary outcomes were as follows: GMFM-66, 53.63 ± 11.94 to 54.96 ± 12.31, *p* = 0.04; step length, 0.32 ± 0.16 to 0.34 ± 0.16, *p* = 0.25; 2-MWD, 59.1 ± 57.0 to 62.8 ± 63.3, *p* = 0.54; COPM performance score, 3.7 ± 2.0 to 5.3 ± 1.9, *p* = 0.06; COPM satisfaction score, 3.3 ± 2.1 to 5.1 ± 2.1, *p* = 0.04.

**Discussion:**

In this exploratory study, we applied a new size of wearable cyborg HAL (2S size), to children with central nervous system disorders. We evaluated its safety, feasibility, and identified an optimal assessment method for multiple treatments. All participants completed the protocol with no serious adverse events. This study suggested that the GMFM would be an optimal assessment tool for validation trials of HAL (2S size) treatment in pediatric patients with posture and motor function disorders.

## 1. Introduction

Children with central nervous system disorders, such as cerebral palsy (CP) or spinal cord disorders, possess positive symptoms like spasticity, clonus, and excessive co-contraction as well as negative symptoms such as weakness and sensory deficits (1). Furthermore, their interactions cause musculoskeletal pathologies, such as equinus, muscle shortening, and degenerative arthritis (1). Children with postural and motor function disorders exhibit characteristic postures typified by equinus and scissoring (2). As a result, walking speed and endurance are reduced (3, 4). However, the range of motion and mobility of the lower extremities improve daily activities and social participation (5). Postural disorders and movement impairment caused by CP and spinal cord diseases are unresolved issues that pediatric patients face throughout their lifespan.

The wearable cyborg Hybrid Assistive Limb® (HAL, Cyberdyne, Tsukuba, Japan) is the world’s first cyborg-type wearable robotic device (6, 7). The HAL detects bioelectrical signals (BES) generated during the patient’s muscle movements, floor reaction force signals generated through intentional weight transfer, or both (7). BES, including myoelectric and other signals, are useful and reliable in estimating human motor intentions (7). In addition, the power unit amplifies the user’s joint torque, estimated from the user’s BES to generate power-assisted torque, and the HAL facilitates muscle activity and exercise by providing motion support based on voluntary movement and walking ability (7). Previous reports have revealed effectiveness of HAL treatment after stroke (8), spinal cord injury (9), neuromuscular diseases (10), and orthopedic surgery (11). Recent studies have also revealed that adolescents with CP showed an immediate improvement in walking speed, step length, and lower limb joint angles during gait after a single session of HAL treatment (12, 13). Furthermore, regarding the effectiveness of multiple HAL treatment, Ueno et al. reported improvements in comfortable walking speed, step length, and cadence after eight sessions of HAL treatment (14), while Matsuda et al. reported improvements in walking speed, single leg support rate, lower limb joint angle during gait, 6-min walking distance (6-MWD), and gross motor function measures (GMFM) (15). Thus, the effectiveness of HAL treatment in adolescents with CP has been demonstrated. However, the appropriate height required for the HAL is >150 cm; therefore, it cannot be used for pediatric patients.

Consequently, a new smaller-sized HAL (2S size, HAL-FC01, Cyberdyne, Tsukuba Japan) was developed for use in patients with postural and motor function disorders. Nakagawa et al. reported an immediate effect and safety profile of single-session treatment with the smaller HAL, and confirmed significant improvements in walking speed, step length, and joint angles of the lower limbs (16). In addition, Kuroda et al. reported a case of a patient with CP, in which the walking speed, 6-MWD, GMFM, and Canadian Occupational Performance Measure (COPM) were improved, and the effects on walking speed and GMFM sustained until the 3-month follow-up (15). However, to our knowledge, there have been no reports verifying multiple sessions of HAL (2S size) treatments in pediatric patients. Based on the past studies, we planned an exploratory clinical study of multiple treatment using the HAL (2S size) to examine the feasibility and safety of the protocol regarding number of treatment sessions, duration, and content of treatment, and to explore the optimal evaluation measures to be used in validation trials of HAL (2S size) in pediatric patients.

## 2. Materials and methods

### 2.1. Participants

We conducted a single-arm study to compare the effects of HAL (2S size) before and after treatment in children with postural and motor function disorders, including CP, spina bifida, cerebrovascular disorders, and cerebrospinal cord injuries. Because this study was designed as an exploratory study to test the efficacy of HAL in pediatric patients with brain and spinal cord disorders, patients with multiple disorders were included. The inclusion criteria were as follows: (1) written consent provided by their substitute; (2) aged 5–15 years; (3) classified as level II–IV on the gross motor function classification system (GMFCS); (4) at least 1 year passed since the disease onset; (5) compatible with the HAL (2S size) device; and (6) willing for continuous hospitalization or outpatient visits according to the study schedule during the study period. The GMFCS classifies the severity of gross motor function impairment in children with disabilities (17). The GMFCS was developed to evaluate CP, but has also been used clinically for other pediatric disorders such as spina bifida (18). The exclusion criteria included the following: (1) difficulty performing voluntary limb movements as instructed due to impaired consciousness or severe intellectual disability; (2) severe skeletal deformities such as osteoarthritis, spondyloarthropathy, or scoliosis that would make training involving joint movements or wearing HAL (2S size) difficult; (3) training problems due to bleeding tendency or osteoporosis; (4) inability to have HAL bioelectrodes applied due to skin diseases; (5) participation in other studies within 12 weeks of the start of this clinical study; and (6) participation deemed inappropriate by the physicians and physical therapists.

Subjects were recruited at each site where the study was conducted, and consent was obtained from both family members and subjects using an explanatory document.

### 2.2. HAL treatment

**Figure 1:**
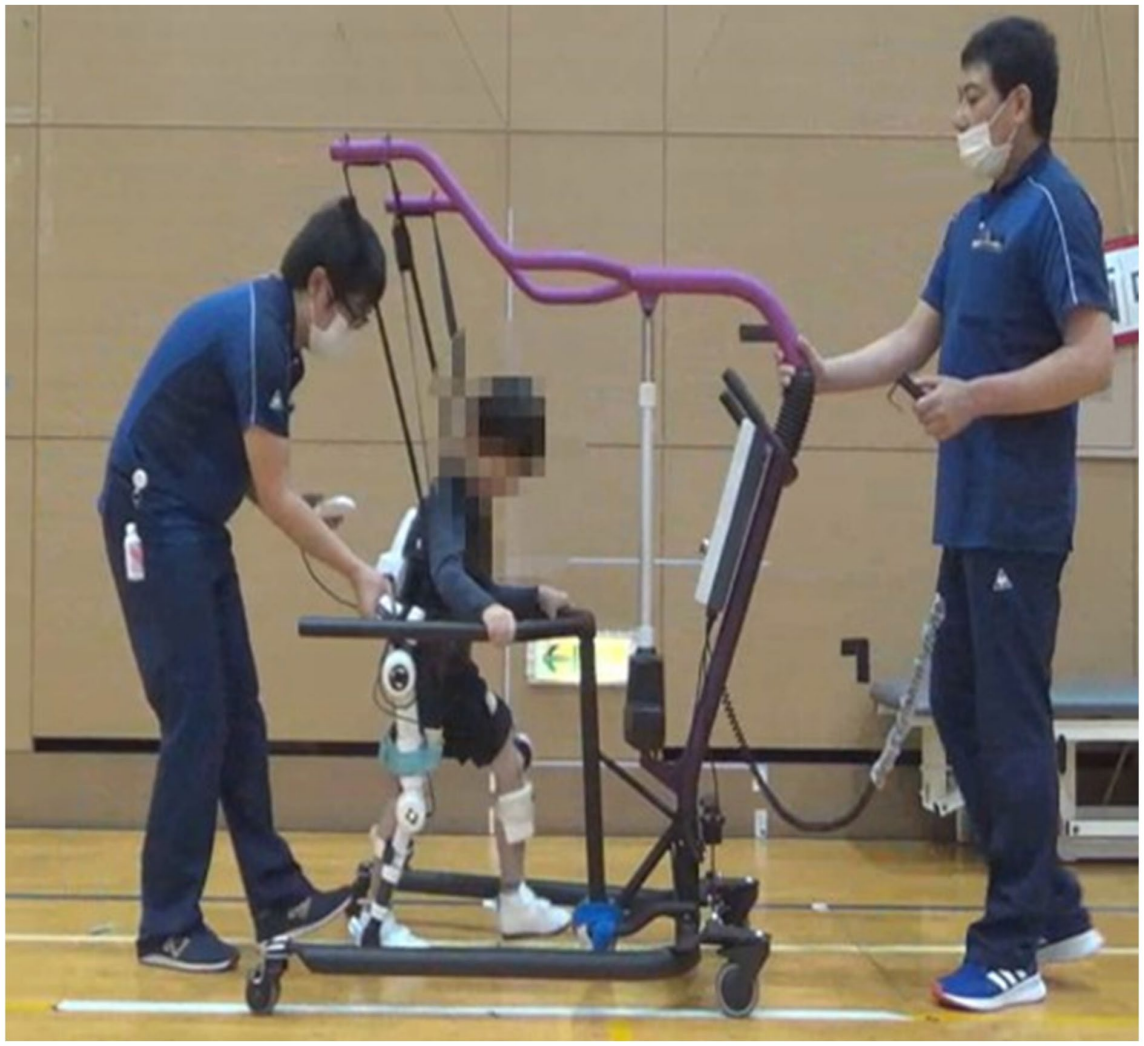
HAL (2S size) treatment.

In this study, HAL (2S size) was used (Figure 1). The control mode of the HAL (2S size) was set to the Cybernic Voluntary Control (CVC) mode, which controls the assist torque based on the intensity of the BES (7). The CVC mode measures the BES from electrodes attached to the hip joint and knee joint flexor and extensor muscles and adjusts the assist torque accordingly. To ensure safety, the assist can be set to a torque limit and an assist angle range limit, combined according to the physical functions of each patient. Assist torque was initially set low and gradually increased as the subject became accustomed to the assist.

The HAL (2S size) treatment was performed once a day for 20 min for a total of 8–12 sessions, during a 5–8 weeks intervention period. It consisted of knee flexion/extension training, stand-up training, standing, and gait training; warm-up and cool-down were performed before and after treatment, respectively, as well as the outcome measures. Assessments at the beginning were made 1 week before the treatment period and assessments at the end were made 1 week after the end of treatment. This study was conducted from July 2020 to July 2021 at the University of Tsukuba Hospital, Ibaraki Prefectural University of Health Sciences Hospital, and the National Hospital Organization Niigata National Hospital. This study was conducted in an outpatient or inpatient setting. Patients who lived too far from the study location or were unable to participate as outpatients were admitted to take part in the study.

### 2.3. Outcome measures

The primary outcome measure was GMFM-88 score, the gold-standard assessment of gross motor function in children. The evaluation items covered five fields: (A) lying down and rolling; (B) sitting; (C) crawling on hands and knees, and kneeling; (D) standing; and (E) walking, running, and jumping (19). The GMFM score was determined an independent third-party video evaluator. The GMFM was developed to evaluate CP (19), but has also been used clinically for other pediatric disorders such as spina bifida (20). The secondary outcome measures included the GMFM-66, 10-m walk test (10-MWT), 2-MWD, 6-MWD, COPM performance and satisfaction, occurrence of disease and test equipment failure. In addition, a questionnaire was administered after HAL (2S size) treatment.

The GMFM-66 is an interval scale version of the GMFM-88, an ordinal scale, reduced to 66 items by Rasch analysis for easy evaluation. By inputting the results of the 66 items into a dedicated program, the GMFM-66 score is calculated; this indicates the gross motor function of children with CP in relation to a 5-year-old child with typical development set at 100 (21). The COPM is a 10-point scale that evaluates a patient’s performance and satisfaction with task goals and assesses life functions (22). COPM questionnaires were administered to participants and their parents at the end of the study. The scale for questionnaires was an 11-point scale including surveys on the level of satisfaction with HAL.

### 2.4. Statistical analyses

Statistical analyses were performed using paired samples t-test or Wilcoxon signed-rank test, depending on the distribution of the data. Two-sided *p* < 0.05 was considered statistically significant with a confidence coefficient of 95%. All analyses were performed using R software.

The study protocol was designed according to the Declaration of Helsinki and relevant ethical guidelines for clinical research. This study was approved by the Tsukuba University Clinical Research Review Board (TCRB19-025) and the Ibaraki Prefectural University of Health Sciences (e261). The study protocol was approved by the Japan Registry of Clinical Trials (jRCTs032200037).

## 3. Results

**Figure 2:**
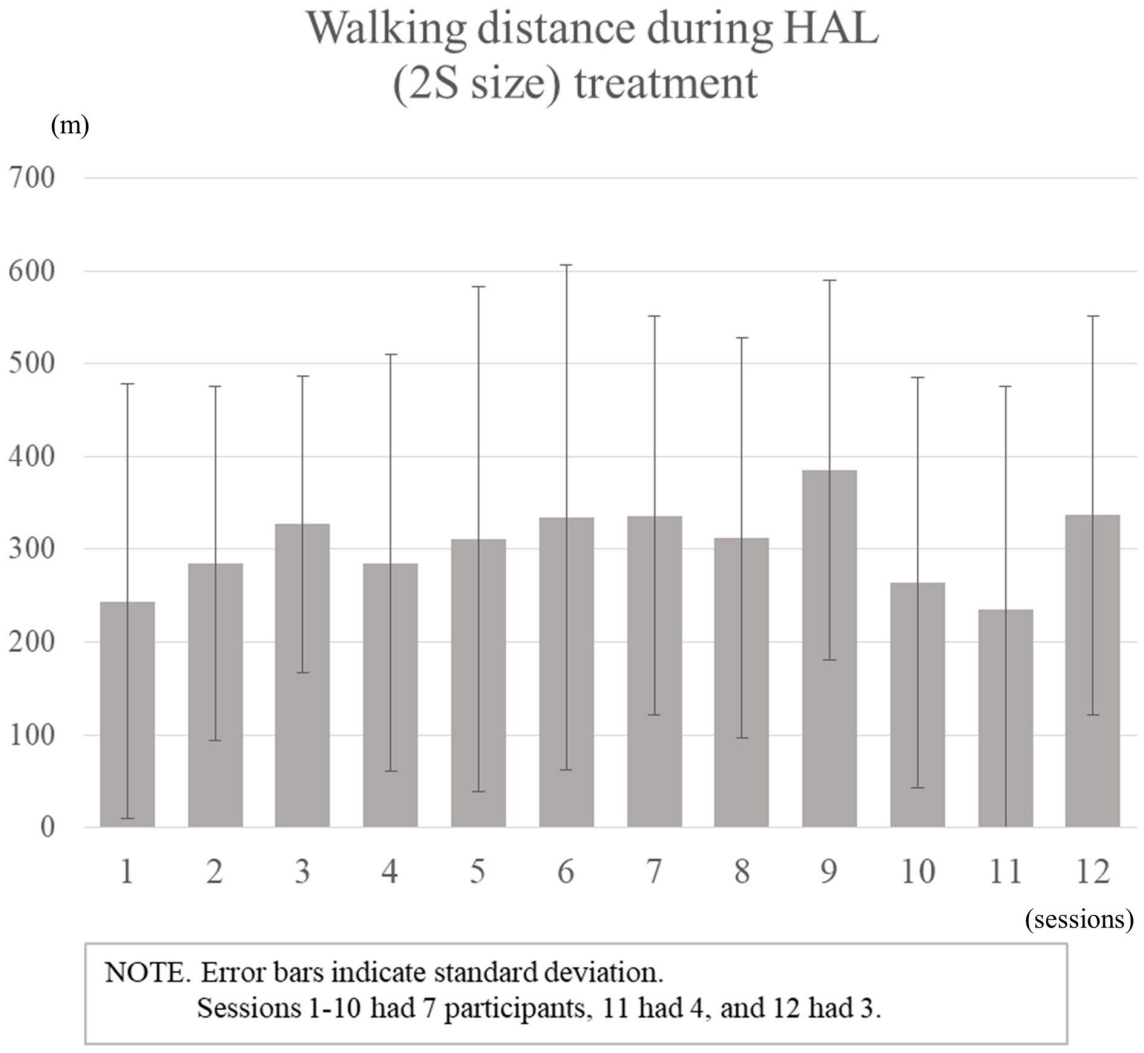
Walking distance during HAL (2S size) treatment.

Seven patients participated in and completed the study protocol. No children were excluded because of their inability to train for HAL. There were three boys and four girls between the age of 6–10 years (mean ± standard deviation, 8.4 ± 1.4), with mean height 120.1 ± 7.8 cm (range, 107.0–127.5 cm) and weight of 25.9 ± 8.8 kg (range, 16.2–39.4 kg). Five were diagnosed with CP and two with spinal cord disorders. The severity of mobility was GMFCS level II in two patients, III in two, and IV in three. Three patients participated in the study as outpatients, whereas the other four were hospitalized. The details of the patients are presented in Table 1. The average number of HAL (2S size) treatment sessions was 11 (range, 10–12), the average walking time was 15 min 4 s, and the average walking distance was 305.7 m (Figure 2). Device failure events were cable damage and thigh cuff damage, both of which were minor. Further, adverse events included erythema of the foot and thigh and skin exfoliation of the feet in Case 2, and myalgia of the lower limbs in Case 3, which were also minor and easily resolved.

**Table 1:** Participant characteristics.

No.	Age (y)	Sex	Height (cm)	Weight (kg)	Diagnosis	Disability	GMFCS
1	8	B	122	28.0	PVL	Spastic/Diplegia	III
2	6	G	107	16.2	PVL	Spastic/Diplegia	III
3	8	G	112	19.2	Ewing sarcoma of the spine	Hypotonia/Paraplegia	II
4	8	B	121	21.8	HIE	Ataxia/Diplegia	II
5	9	G	128	35.6	PVL	Spastic/Quadriplegia	IV
6	10	B	126	20.8	PVL	Mixed/Quadriplegia	IV
7	10	G	126	39.4	Spina bifida	Hypotonia/Paraplegia	IV

B, Boy; G, Girl; PVL, Periventricular Leukomalacia; HIE, Hypoxic Ischemic Encephalopathy; GMFCS, Gross Motor Function Classification System.

The paired samples t-test was performed for the GMFM-88, 10-MWT, 2-MWD, GMFM-66, and COPM. The Wilcoxon signed-rank test was performed for the 6-MWD. GMFM-88 score, the primary outcome measure, improved from 65.51 ± 21.97% to 66.72 ± 22.28%, *p* = 0.07. The changes in secondary outcome measures after the treatment were as follows: GMFM-66, 53.63 ± 11.94 to 54.96 ± 12.31, *p* = 0.04; step length, 0.32 ± 0.16 to 0.34 ± 0.16, *p* = 0.25; 2-MWD, 59.1 ± 57.0 to 62.8 ± 63.3, *p* = 0.54; COPM performance score, 3.7 ± 2.0 to 5.3 ± 1.9, *p* = 0.06; COPM satisfaction score, 3.3 ± 2.1 to 5.1 ± 2.1, *p* = 0.04 (Table 2).

**Table 2:** Assessment results before and after HAL (2S size) treatment.

			Before-HAL	After-HAL	*p*-value
**Primary outcome**					
GMFM-88	Total	(%)	65.51 ± 21.97	66.72 ± 22.28	0.07
**Secondary outcome**					
GMFM-66		(score)	53.63 ± 11.94	54.96 ± 12.31	0.04
10 m-walk test	Speed	(m/s)	0.65 ± 0.54	0.64 ± 0.50	0.96
	Stride	(m)	0.32 ± 0.16	0.34 ± 0.16	0.25
	Cadence	(steps/min)	99.33 ± 53.99	97.77 ± 45.26	0.87
2-min walk distance		(m)	59.1 ± 57.0	62.8 ± 63.3	0.54
6-min walk distance		(m)	115.2(50.0–188.2)	87.0(65.3–213.3)	0.69
COPM	Performance average	(score)	3.7 ± 2.0	5.3 ± 1.9	0.06
	Satisfaction average	(score)	3.3 ± 2.1	5.1 ± 2.1	0.04
**Secondary analysis**					
GMFM-88	Dimensions D (Standing)	(%)	41.03 ± 32.37	44.69 ± 36.62	0.17
	Dimensions E (Walking, running, jumping)	(%)	29.96 ± 30.92	30.75 ± 30.00	0.55
	Additive mean of D and E	(%)	35.50 ± 31.37	37.72 ± 33.21	0.12
COPM	Revision performance average	(score)	2.0 (1.0–3.5)	6.0 (1.0–8.0)	<0.01
	Revision satisfaction average	(score)	2.0 (1.0–3.5)	6.0 (1.0–8.5)	0.01

GMFM: Gross Motor Function Measure, COPM: Canadian Occupational Performance Measure. Values are mean ± SD or median (quartile range).

Our findings indicated that HAL treatment contributed to improvements in standing and walking function. However, GMFM-88 and GMFM-66 are comprehensive gross motor function assessments and cannot specifically identify changes in standing or walking function. Therefore, we conducted a secondary analysis of GMFM dimension D (GMFM-D; standing) (23), GMFM dimension E (GMFM-E; walking, running, jumping) (23), and the additive mean of GMFM D and E (GMFM-D + E) (24). The secondary analysis revealed improvement in scores after the treatment, as follows: GMFM-D, 41.03 ± 32.37% to 44.69 ± 63.62%, *p* = 0.17; GMFM-E, 29.96 ± 30.92% to 30.75 ± 30.00%, *p* = 0.55; GMFM-D + E, 35.50 ± 31.37% to 37.72 ± 33.20%, *p* = 0.12 (Table 2). The sample size of the study was small, and there was wide variation in GMFM scores. Therefore, individual GMFM data are presented (Table 3).

**Table 3:** GMFM scores for individual participants.

GMFM-88			GMFM-66			GMFM-D			GMFM-E		
No.	Pre	Post	No.	Pre	Post	No.	Pre	Post	No.	Pre	Post
1	78.35	81.29	1	60.62	63.98	1	56.41	71.79	1	51.39	50.00
2	66.30	65.59	2	52.32	52.62	2	33.33	30.77	2	18.06	19.44
3	91.97	91.77	3	71.69	71.69	3	82.05	87.18	3	77.78	73.61
4	84.97	87.87	4	61.8	64.63	4	79.49	87.18	4	54.17	59.72
5	45.56	46.56	5	43.44	44.03	5	2.56	2.56	5	0.00	0.00
6	30.36	31.09	6	36.79	37.43	6	7.69	7.69	6	0.00	0.00
7	61.05	62.84	7	48.73	50.32	7	25.64	25.64	7	8.33	12.50

GMFM, Gross Motor Function Measure.

Owing to the nature of COPM, which extracts subjective work goals from patients, evaluators should not intentionally guide goal setting. Therefore, the results of the original COPM showed that there were many goals that may not relate to HAL (2S size) treatment. Thus, we performed a secondary analysis with the goals only related to Wilcoxon’s signed rank-test (Table 4). COPM performance and satisfaction improved significantly from 2.0 (quartile range 1.0–3.5) to 6.0 (1.5–8.0) and from 2.0 (1.0–3.5) to 6.0 (1.0–8.5), respectively (performance *p* = 0.008, satisfaction *p* = 0.012; Table 2).

**Table 4:** Example of drafted goals of the COPM.

Example of included goals	Example of excluded goals
I want to walk safely with a cane.	I want to be able to eat by myself.
I want to be able to stand safely without braces.	I want to learn more letters.
I want to go up and down stairs safely.	I want to get better at changing clothes.
I want to be able to hold on when transferring to the toilet.	I want to be better at wheelchair basketball.
I would like to be able to go in and out of the bathroom by myself.	I want to play the piano better.
(Total 19 goals)	(Total 16 goals)

Furthermore, the questionnaires indicated a high level of satisfaction with a score of 9.1 for the question “Are you satisfied with HAL (2S size-prototype)?” and 9.0 for “Are you satisfied with your HAL (2S size) treatment?.” Participant’s parents had scores of 9.1 and 9.3, respectively, for the same questions indicating that they were also highly satisfied (Table 5). Additionally, free comments from the participants included “The HAL (2S size) treatment was fun” and “The walking test increased.” Free comments from the parents included “Decreased assistance with toileting of my child” and “I think my child is getting in and out of the car better.” Additionallu, there were positive comments about the improvement in daily life activities (Table 5).

**Table 5:** Questionnaire results for HAL (2S size) treatment.

Participants (*n* = 7)		Mean value
Are you satisfied with HAL?	0【unsatisfactory】−10【Satisfactory】	9.1
Are you satisfied with your HAL treatment?	0【unsatisfactory】−10【Satisfactory】	9.0
Did you feel pain or distress?	0【None】 −10【Severe pain】	0.7
**Parents (*n* = 7)**		**Mean value**
Are you satisfied with HAL?	0【unsatisfactory】−10【Satisfactory】	9.1
Are you satisfied with participant’s HAL treatment?	0【unsatisfactory】−10【Satisfactory】	9.3
Do you feel that the participant moves better in everyday life?	0【unsatisfactory】−10【Satisfactory】	8.7
Do you feel that participants’ spasticity has been reduced?	0【unsatisfactory】−10【Satisfactory】	7.1
Do you feel that the participant’s caregiving needs have been reduced?	0【unsatisfactory】−10【Satisfactory】	7.3
**Examples of participant’s free comments**		
The HAL treatment was fun.	HAL treatment has improved lower limb movement.	
The walking distance increased.	The HAL felt heavy.	
I felt that HAL treatment helped stabilize my core.	There was pain when I wore the HAL.	
Example of parents free comment		
Decreased assistance with toileting of my child.	I would like to see HAL treatment done more often.	
I think my child is getting in and out of the car better.	I want HAL to have the ability to move autonomously and not fall down.	
The child’s walking distance was extended.		

## 4. Discussion

This study was conducted to examine the feasibility and safety of HAL (size 2S) treatment and to identify the optimal assessment index. The absence of serious adverse events was comparable to previous studies that evaluated the safety of the HAL (2S size) in pediatric patients with postural and motor function disorders (13, 14). We believe that our HAL treatment protocol carries low risk and can be considered safe for HAL intervention. The secondary aim of this study was to investigate the feasibility of a HAL (2S size) for future large-scale studies. The study protocol, based on previous research, stipulated that HAL (2S size) treatment should be performed once a day for 20 min for a total of 8–12 sessions (10, 14, 25). The average number of the sessions of HAL (2S size) treatment was 11 (range, 10–12). Since the patients were children, we were concerned about a high dropout rate due to mood swings or lack of concentration; however, all patients exceeded the prescribed number of sessions.

The primary outcome measure, the GMFM-88, did not show any statistically significant improvements.

In recent years, the Minimal Clinically Important Difference (MCID) has gained attention as a standard method for measuring clinical appropriateness and to determine the quality of treatment (26). The MCID score is defined as the minimal amount of change that is important to the patient and can be used to establish therapeutic thresholds in outcome measures (26). Storm et al. reported an MCID of 0.1%–3.0% for GMFM-88 (27). The change in this study was 1.21%, which was partially higher than the MCID score. Further, GMFM-66, a secondary outcome measure, showed significant improvement. Oeffinger et al. reported an MCID of 0.8–1.3 for GMFM-66 (28). The change in this study was 1.3, which was partially higher than the MCID. In addition, a secondary analysis was performed on GMFM-D, GMFM-E, and GMFM-D + E, and the results showed no statistically significant improvement. However, in our study, the change in GMFM-D was 3.06% and change in GMFM-E was 0.79%, comparable to the respective MCID values of 0.8–5.2% and 0.3–4.9% reported by Storm et al. (27). HAL is a robotic device that functions as if it was part of the body according to the user’s motor intentions and ideal internal movement patterns. Training with HAL allows the user to actively participate and practice adapting dynamic gait patterns, which is a necessary component for improved motor learning. We believe that motor learning acquired with the HAL influences motor function after the HAL is removed. In addition, the effects of HAL have been reported to persist even after 1 year (29).

In terms of feasibility, this study included only a small number of patients (n = 7), involving three children with severe disorders with GMFCS level IV, which made it difficult to improve the GMFM (30). Subjects with GMFCS level IV (No 5, 6, and 7) did not improve their scores on the GMFM-D. Additionally, the GMFM-E scores of Case No. 5 and 6 were zero, indicating a floor effect (Table 3). These factors may have prevented statistically significant changes in GMFM-88, GMFM-D, GMFM-E and GMFM-D + E. However, the changes in GMFM was partially higher than that in the MCID, and we considered that there was a clinically meaningful improvement. We hypothesized that GMFM, which can assess standing and walking ability, would be a valid assessment index for HAL treatment.

Regarding walking ability, the results of the 10-MWT, 2-MWD, and 6-MWD improved from the first to the final assessment, but the differences were not significant. Thompson et al. reported that the intra-class correlation coefficient for the 10-MWT for children with CP ranged from 0.58 to 0.78 and the minimum detectable change ranged from 1.7 to 12.2 s, an assessment with a large range of measurement error (4). For others, performance for 6-MWD decreased without continuous cheers and was highly dependent on the child’s level of understanding and motivation (31). In addition, gait performance decreased with tiredness after the 6-MWD in children with CP who were able to walk (3). Several pilot studies of HAL (2S size) treatment in children have demonstrated improvement, primarily through gait assessments, such as the 10-MWT and 6-MWD, and gross motor assessments, such as the GMFM[13; 14; 15; 16; 25]. However, based on this study, gross motor assessments may be more suitable for assessing the effectiveness of HAL than walking assessments.

Furthermore, an increase in COPM satisfaction was observed. In pediatric robot-assisted gait training studies, it is important to reflect on the rehabilitation goals of individual children in the assessment measures. COPM is a valid assessment tool (32); we found it clinically significant that the COPM results demonstrated high satisfaction with the work goals set by the patients and their parents.

This study has a few limitations. First, it was conducted using a single-arm design with patients evaluated before and after the intervention and without a control condition, making it susceptible to confounding factors. Second, the statistical power was low due to the small sample size. Nevertheless, this study was intended as a preliminary study for larger studies to be conducted in the future. The results of this study will be used to develop a protocol for a randomized controlled trial with the presence of a control group.

## 5. Conclusion

In this study, we applied a new size of the wearable cyborg HAL (2S size) to children with central nervous system disorders, and examined its safety, feasibility, and optimal assessment for multiple treatments. All participants completed the protocol with no serious adverse events. Additionally, the GMFM was determined to be the optimal assessment tool to evaluate the HAL treatment.

## Data availability statement

The raw data supporting the conclusions of this article will be made available by the authors, without undue reservation.

## Ethics statement

The studies involving humans were approved by Tsukuba University Clinical Research Review Board and the Ibaraki Prefectural University of Health Sciences. The studies were conducted in accordance with the local legislation and institutional requirements. Written informed consent for participation in this study was provided by the participants’ legal guardians/next of kin. Written informed consent was obtained from the individual(s) for the publication of any identifiable images or data included in this article.

## Author contributions

KTa: Data curation, Formal Analysis, Investigation, Methodology, Validation, Visualization, Writing – original draft. MM: Conceptualization, Funding acquisition, Investigation, Methodology, Writing – review & editing. HW: Conceptualization, Data curation, Formal Analysis, Investigation, Methodology, Project administration, Validation, Writing – review & editing. MK: Conceptualization, Investigation, Methodology, Writing – review & editing. YSh: Conceptualization, Funding acquisition, Investigation, Methodology, Resources, Writing – review & editing. TaN: Conceptualization, Funding acquisition, Investigation, Methodology, Resources, Writing – review & editing. HM: Resources, Writing – review & editing, Funding acquisition, Investigation. HKam: Conceptualization, Funding acquisition, Writing – review & editing. KTo: Investigation, Writing – review & editing. YH: Conceptualization, Funding acquisition, Resources, Writing – review & editing. KK: Investigation, Methodology, Validation, Writing – review & editing. KY: Investigation, Methodology, Writing – review & editing. ToN: Investigation, Resources, Writing – review & editing. NI: Conceptualization, Funding acquisition, Investigation, Resources, Writing – review & editing. HKaw: Conceptualization, Funding acquisition, Writing – review & editing. YSa: Conceptualization, Funding acquisition, Writing – review & editing. MY: Conceptualization, Funding acquisition, Writing – review & editing. AkM: Conceptualization, Funding acquisition, Writing – review & editing. AiM: Conceptualization, Funding acquisition, Investigation, Methodology, Project administration, Resources, Supervision, Writing – review & editing.
